# Prevalence and epidemiological characteristics of asymptomatic malaria in Sucre, Venezuela: a 2022 cross-sectional study

**DOI:** 10.1186/s12936-025-05356-z

**Published:** 2025-04-13

**Authors:** Jessica L. Leyva, Paola A. Pereira-Leitao, Gabriel E. García-Meléndez, Samuel De Amicis, Rodrigo Celis, Mariana Hidalgo, Antonio Hernández, Fhabián S. Carrión-Nessi, David A. Forero-Peña

**Affiliations:** 1 “Luis Razetti” School of Medicine, Universidad Central de Venezuela, Caracas, Venezuela; 2 Biomedical Research and Therapeutic Vaccines Institute, Ciudad Bolívar, Venezuela; 3 Laboratory of Immunoparasitology, Centro de Microbiología y Biología Celular, Instituto Venezolano de Investigaciones Científicas, Altos de Pipe, Venezuela; 4 Immunogenetics Section, Laboratory of Pathophysiology, Centro de Medicina Experimental “Miguel Layrisse”, Instituto Venezolano de Investigaciones Científicas, Altos de Pipe, Venezuela; 5 Department of Infectious Diseases, Hospital Universitario de Caracas, Caracas, Venezuela

**Keywords:** Prevalence, Epidemiology, Malaria, Asymptomatic Infections, Venezuela

## Abstract

**Background:**

Despite a significant reduction in malaria cases in America, Venezuela has experienced a substantial increase between 2000 and 2019. Asymptomatic malaria, prevalent in both low- and high-endemic regions, poses a challenge due to the absence of clinical manifestations and often low parasitaemia. This study aims to determine the current prevalence of asymptomatic malaria in four rural communities of Sucre, the third most endemic state in the country.

**Methods:**

A community-based cross-sectional study was conducted from October to December 2022 (high seasonality period). Individuals were interviewed in their households and assessed for malaria using rapid diagnostic tests (RDTs), thick and thin blood smear microscopy, and polymerase chain reaction (PCR). Asymptomatic individuals with PCR positive (PCR+) for *Plasmodium* were classified as cases, while PCR negative individuals were classified as controls. Descriptive statistics were used to analyse the data. The normality of numerical variables was assessed with the Kolmogorov–Smirnov test. Based on this assessment, Student’s *t*-test was applied to normally distributed variables and Mann–Whitney U-test to non-normally distributed ones. For categorical variables, Pearson’s chi-square test was used when less than 25.0% of cells had an expected frequency below five; otherwise, Fisher’s exact test was employed.

**Results:**

The study involved 351 individuals, mostly women (54.7%), of mixed (non-indigenous) race (61.3%), with primary (6 years) education (40.7%). The most common occupations were students (30.5%), housekeepers (27.6%), and farmers (16.5%). Over half (54.4%) had lived at their current address for over 10 years. The prevalence of asymptomatic malaria by RDTs and microscopy was 0.3% (*n* = 1/351) as determined. However, PCR detected a higher prevalence of 24.8% (87 positive cases, 95.0% CI = 20.5–29.5), primarily caused by *P. vivax* (73.6%). The highest prevalences were observed in individuals aged over 15 years (27.1%, 95.0% CI = 21.6–33.1), males (28.3%, 95.0% CI = 21.7–35.6), those with a college (14 years) education (33.3%, 95.0% CI = 17.2–53.2), and educators (41.7%, 95.0% CI = 18–68.8). The rural community with the highest prevalence was Chacopata (30.6%, 95.0% CI = 17.4–46.7), followed by El Paujil (28.6%, 95.0% CI = 21.9–36.1), Yaguaraparo (23.2%, 95.0% CI = 15.1–33.1), and Cristóbal Colón (16.5%, 95.0% CI = 9.6–25.8). Two-thirds (66.7%) reported a malaria history, predominantly caused by *P. vivax* (70.5%), with a median of 3 previous episodes. At least one-third (35.5%) had non-adherence to treatment during their most recent malarial episode. No statistically significant differences were observed between sociodemographic characteristics and malaria history of individuals with asymptomatic malaria (PCR+) and controls.

**Conclusion:**

RDTs and microscopy only managed to diagnose less than 1.0% of asymptomatic malaria cases. Active surveillance systems with high sensitivity such as PCR may provide accurate estimates of asymptomatic malaria prevalence needed for opportune diagnosis and treatment.

**Supplementary Information:**

The online version contains supplementary material available at 10.1186/s12936-025-05356-z.

## Background

The incidence of malaria cases in America has experienced a significant reduction of 73.7%, from 13.5 to 3.6 cases per 1,000 population at risk, between 2000 and 2023. However, this progress has been hindered in recent years due to a substantial increase in malaria cases in Venezuela, which escalated from approximately 35,500 cases in 2000 to over 467,000 in 2019 [1]. Despite the COVID- 19 pandemic leading to a gradual decrease in malaria cases in Venezuela, from 223,000 cases in 2020 to 135,000 in 2023, Venezuela is now ranked the country with the second highest notified cases in the region. Together with Brazil and Colombia, these countries account for 76,8% of all cases in the region [1].

From 1999 to 2002, Sucre was recognized as the Venezuelan state with the highest prevalence of malaria [2]. Specifically, Cajigal municipality had the highest annual parasite index (API) in the country, with 260 cases per 1,000 inhabitants. Together with Benítez municipality, these areas are considered potential epicentres for epidemic outbreaks [3]. Currently, Sucre, Bolívar, and Amazonas are the three states that contribute to more than 90.0% of malaria cases in Venezuela [4]. Unlike the other two states, the transmission foci in Sucre are primarily associated with agricultural activities, unfavourable socioeconomic conditions, and unplanned urban growth [5].

According to Epidemiological Bulletin 2022 from Venezuelan Ministry of Health (Epidemiological Week No. 41) [4], the most recent official report on the epidemiological status of malaria in Venezuela, Sucre accounted for 13.0% (*n* = 13,140) of the national cases, ranking as the third most endemic state in the country, after Bolívar (59.4%, *n* = 60,329) and Amazonas (18.3%, *n* = 18,580). The API in Sucre was 14.7 per 1,000 inhabitants. *Plasmodium vivax* accounted for 92.8% of cases, followed by *Plasmodium falciparum* with 3.4% and mixed *P. vivax*/*P. falciparum* infection with 3.8%. Sucre (*n* = 5,683), Cajigal (*n* = 1,987), and Ribero (*n* = 1,394) municipalities reported the highest cumulative cases numbers to date [4].

Asymptomatic malaria is prevalent in both low- and high-endemic regions, constituting important reservoirs for the parasite and contributing to malaria transmission [6–11]. The diagnosis of asymptomatic malaria is complex due to the absence of clinical manifestations and often low parasitaemia. Passive surveillance, and even mass screening and treatment (MSAT) with rapid diagnostic tests (RDTs) and microscopy techniques, are ineffective in detecting asymptomatic cases, with sensitivity ranging from 25.0–60.0% [12–21]. The limit of detection of parasites for RDTs (100 parasites/µL) [22] and microscopy (50–500 parasites/µL) [23] precludes the detection of low parasitaemia. In contrast, molecular techniques such as polymerase chain reaction (PCR) (1–5 parasites/µL) [24] provide accurate estimates of the prevalence of asymptomatic malaria. Most studies of asymptomatic malaria report *P. falciparum*. However, in areas of high endemicity, *P. vivax* may be found in asymptomatic individuals, which has earlier gametocyte emission and thus earlier transmission (compared to other species) [2, 20]. This highlights the fundamental role of molecular diagnostics in understanding the epidemiology of malaria.

As the global public health goals for malaria shift from disease control to elimination, there is growing interest in the importance of asymptomatic infections and the optimal diagnostic tests to identify them. However, studies of asymptomatic malaria in Venezuela are scarce. In Sucre, a prevalence of asymptomatic malaria of 8.0% was reported by PCR in Cajigal in 2003 [2]. There have been no studies investigating the prevalence of asymptomatic individuals in Venezuela in the last 20 years. This study aims to determine the current prevalence of asymptomatic malaria in four communities of Sucre state using molecular techniques.

## Methods

### Study area

This study was conducted in Sucre state, located in the northeastern region of Venezuela. Covering an area of approximately 11,800 km^2^, Sucre has a population of more than 1,087,779, distributed across 15 municipalities and 56 parishes [25].

The incidence of malaria in this region is subject to fluctuations, largely influenced by the diverse climatic conditions of the state, which include both coastal and mountainous areas. The transmission of malaria typically escalates during the rainy season (May to November), and recedes during the drier months [26].

Data collection for this study occurred from October to December 2022, coinciding with a period of high transmission due to rainy conditions. Sucre’s geography, characterized by its low altitude and steep slopes, provides favourable conditions for the formation of wetlands, thereby promoting the proliferation of breeding sites for *Anopheles aquasalis* [27].

**Fig. 1:**
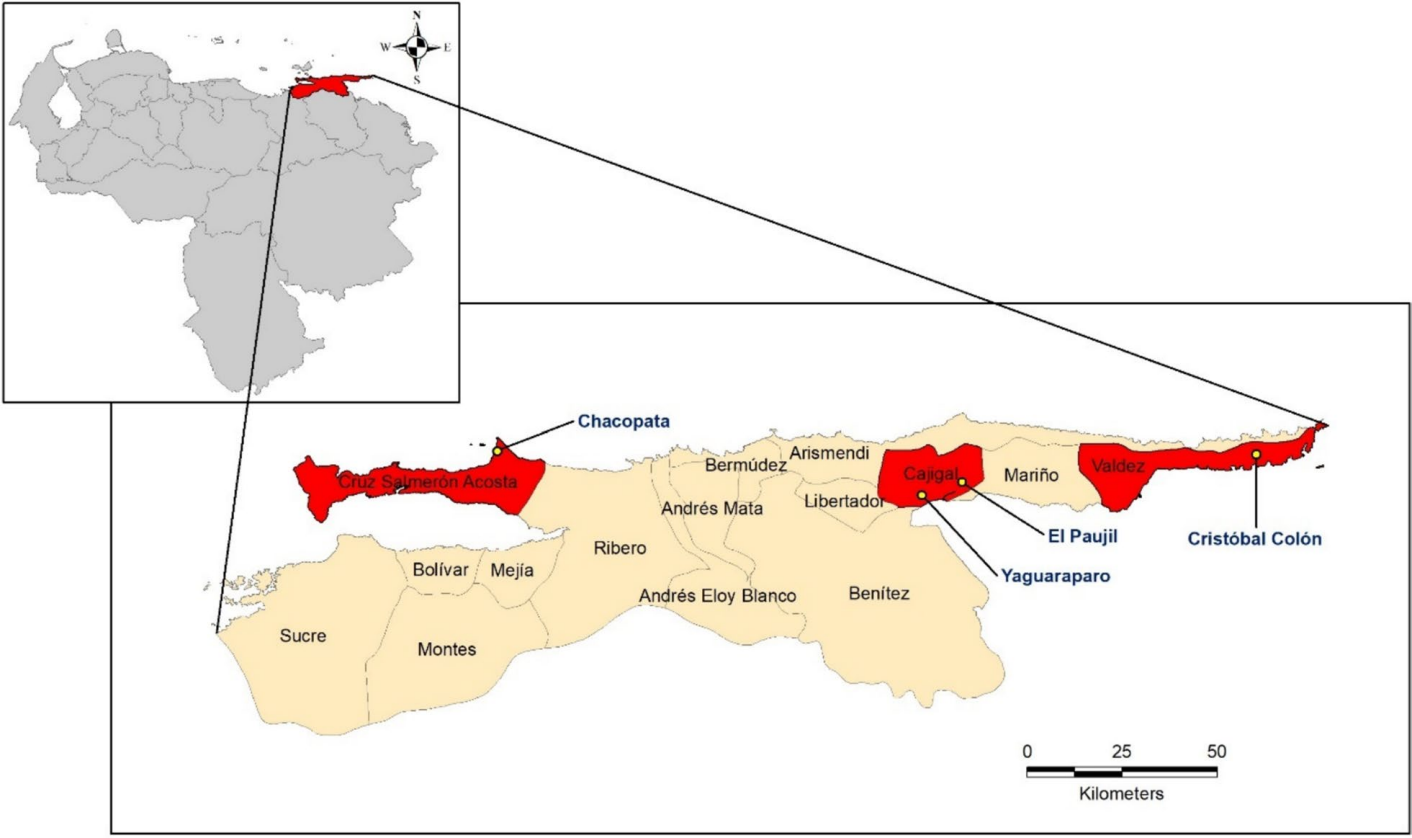
Map of Sucre state, its municipalities, and the four selected rural communities

**Fig. 2:**
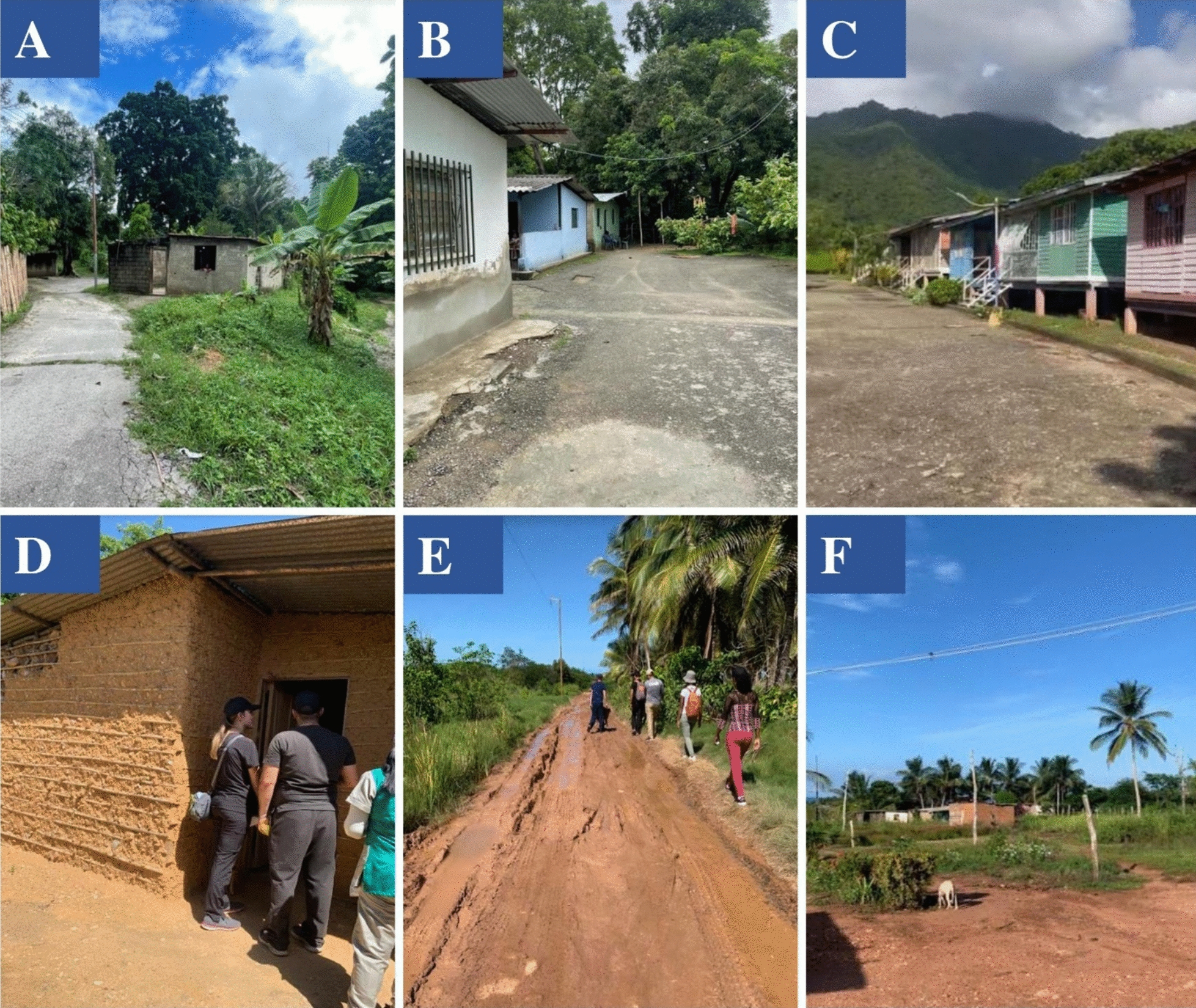
Characteristics of the houses in the four rural communities included in the study.** A** Yaguaraparo (Cajigal municipality): houses with block walls and zinc roofs. **B** El Paujil (Cajigal municipality): houses with cement walls and zinc roofs. **C** Cristóbal Colón (Valdez municipality): wooden houses. **D** El Paujil (Cajigal municipality): houses with mud walls and zinc roofs. **E** Chacopata (Cruz Salmerón Acosta municipality): dirty roads in the community. **F** Chacopata (Cruz Salmerón Acosta municipality): mud houses with zinc roofs

This study was conducted in four rural communities (parishes): Chacopata, Yaguaraparo, El Paujil, and Cristóbal Colón, which belong to the jurisdiction of three municipalities: Cajigal (Yaguaraparo and El Paujil), Valdez (Cristóbal Colón), and Cruz Salmerón Acosta (Chacopata) (Fig. 1). The housing structures in these communities varied in characteristics, ranging from mud-brick houses with dirty floors to block houses with cement floors (Fig. 2).

### Study design and population

A community-based cross-sectional study was conducted from October to December 2022. The study population consisted of all individuals residing in the four selected rural communities of Sucre state. Race was categorized as Black, White, and Mixed (defined as “mestizo” or “mulatto”, referring to individuals of European, Indigenous, and/or African ancestry, a classification common in Latin America).

Exclusion criteria included: (1) exhibiting malaria symptoms or signs within the last 48 h, (2) having a tympanic temperature > 38 °C, (3) not being a resident (defined as living in one of the selected communities for at least six months before the study), and (4) taking anti-malarial drugs within the four weeks preceding recruitment.

### Sampling method

The sample size was calculated using the single population proportion sample size calculation formula, with a confidence level of 95.0%, a margin of error of 5.0%, and a proportion of asymptomatic malaria among individuals in Sucre state of 8.0% [2]. The final calculated sample size was 114. The study employed a systematic sampling method, involving door-to-door visits to each household in the village for interviews and sample collection. Out of the 15 rural communities in Sucre, four were selected as representative communities using a cluster sampling technique. A total of 504 individuals were initially interviewed and tested using RDTs. Out of these, 109 were excluded due to symptoms, 13 for recent anti-malarial use, and 31 for damaged samples. Ultimately, 351 asymptomatic individuals met the inclusion criteria and were included in the analysis.

### Data collection

Five trained physicians administered a structured, pre-tested questionnaire using EpiCollect as the data collection platform. This questionnaire (Supplementary Data 1) was designed to collect information on the sociodemographic characteristics of the individuals (including age, sex, race, education level, occupation, pregnancy status, current address, and length of residence at current address) and their malaria history (including previous malaria episodes, total number of episodes, parasite identified in the last episode, and adherence to treatment in the last episode).

### Blood sample collection and processing

A total of three capillary blood samples were collected from each individual by earlobe puncture using a disposable lancet. The first blood sample was used for parasitological diagnosis of *Plasmodium* spp. by thick and thin blood smear microscopy, stained with the Giemsa method [28]. These smears were independently examined by two experienced microscopists, who recorded *Plasmodium* species under 1,000X magnification. The second blood sample was used for diagnosis by RDT. All individuals diagnosed with asymptomatic malaria, either by microscopy and/or RDT, were treated by the local health provider, following the most recent national antimalarial protocol, approved in 2017 by the country’s health authorities [29]. The third blood sample was collected on filter paper (Whatman No. 1), dried, stored in a plastic bag with desiccant, and transferred to the Laboratory of Immunoparasitology of the Centro de Microbiología y Biología Celular (Instituto Venezolano de Investigaciones Científicas) for molecular diagnosis of *Plasmodium* spp. by nested PCR [30].

### DNA extraction

DNA extraction from blood spots on filter paper was performed according to the protocol established by Musapa et al. [31]. Briefly, blood spot samples, each with a diameter of 5 mm, were immersed in 1 mL of phosphate-buffered saline. These samples were then incubated at room temperature for 30 min within a 1.5-ml tube, followed by centrifugation at a speed of 7,000 rpm. Post-centrifugation, the supernatant was discarded, and 200 µL of 1.0% Chelex was added. This mixture underwent two stages of incubation: initially at 56 °C for 30 min, and subsequently at 100 °C for 8 min. After these incubation periods, the mixture was centrifuged again at 7,000 rpm for 5 min. The supernatant was then carefully transferred into a 1.5 mL tube and stored at − 80 °C for further analysis.

### PCR for *Plasmodium* species identification

Nested PCR procedure was performed in two stages. The first round of DNA amplification utilized rPLU5 and rPLU6 primers (Supplementary Data 2) to identify the *Plasmodium* genus. The PCR mixture contained 5 µL of VerityMAX™ DNA polymerase master mix [1X], 1 µL of each primer [1 pM], 2 µL of DNA [50–100 ng], and distilled water to complete a final volume of 15 µL. The thermoprofile consisted of an initial denaturation step at 95 °C for 5 min, followed by 35 cycles at 95 °C for 30 s, 55 °C for 1 min and 72 °C for 1 min, and a final extension step at 72 °C for 5 min. Amplicons were subjected to electrophoresis for approximately 40 min at 100 V on 2.0% agarose gels. The expected size was 1200 bp (Supplementary Data 3).

### Nested PCR

All positive samples from the first PCR were subjected to the second PCR round. An aliquot of 2 µL from the product of the first round served as the template for amplification of *Plasmodium* species-specific fragments using three pairs of primers (rFAL-F and rFAL-R, rVIV-F and rVIV-R, rMAL-F and rMAL-R) (Supplementary Data 2). The PCR mixture and thermoprofile were identical to those described in the first PCR round. The PCR products were run on a 2.0% agarose gel for 40 min at 100 V and visualized under an ultraviolet transilluminator. The expected sizes of the PCR products are detailed in Supplementary Data 3.

### Statistical analysis

Asymptomatic individuals with PCR positive (PCR+) for *Plasmodium* were classified as cases, while PCR negative individuals were classified as controls. Individuals’ data were summarized using the following descriptive statistics: mean, standard deviation (SD), median, interquartile range (IQR), and/or frequency, percentage (%). The distribution of numerical variables was assessed using the Kolmogorov–Smirnov test. For numerical variables, Mann–Whitney U test was used for those with a non-normal distribution and Student’s *t* test for independent samples for those with a normal distribution. For categorical variables, Pearson’s chi-square test was used when the expected frequency was less than five in ≤ 25.0% of the cells and Fisher’s exact when the expected frequency was less than five in > 25.0% of the cells. *P* values < 0.05 were considered statistically significant. Statistical analyses were performed using SPSS version 26.

## Results

### Sociodemographic characteristics and history of malaria

The study analysed a total of 351 individuals. The median age of the individuals was 24 (IQR 12–41) years, mostly women (54.7%, *n* = 192), of mixed (non-indigenous) race (61.3%, *n* = 215), and had attained primary (6 years) education (40.7%, *n* = 143). The most common occupations were student (30.5%, *n* = 107), housekeeper (27.6%, *n* = 97), and farmer (16.5%, *n* = 58). More than half of the individuals (54.4%, *n* = 16.5) had resided at their current address for more than 10 years, with a median duration of 12 (IQR 5–22) years. Two-thirds of the individuals (66.7%, *n* = 234) reported a history of malaria, with a median of 3 (IQR 2–7) previous episodes, predominantly caused by *P. vivax* (70.5%, *n* = 165). At least one-third of the individuals (35.5%, *n* = 83) had non-adherence to treatment during their most recent malarial episode (Table 1).

**Table 1:** Sociodemographic characteristics, malaria history, and type of diagnosis of 351 individuals included in the study

	All (*n* = 351, 100.0%)	Prevalence of PCR+	95.0% CI
Age, median (IQR), years	24 (12–41)		
Age group, *n* (%)			
< 5 years	30 (8.5)	5 (16.7)	6.7–32.7
5–15 years	92 (26.2)	20 (21.7)	14.3–31
> 15 years	229 (65.2)	62 (27.1)	21.6–33.1
Sex, *n* (%)			
Female	192 (54.7)	42 (21.9)	16.5–28.1
Male	159 (45.3)	45 (28.3)	21.7–35.6
Race, *n* (%)			
Mestizo	215 (61.3)	52 (24.2)	18.8–30.2
Black	118 (33.6)	29 (24.6)	17.5–32.9
White	18 (5.1)	6 (33.3)	15.3–56.3
Level of education, *n* (%)			
None	80 (22.8)	15 (18.8)	11.4–28.3
Primary school	143 (40.7)	39 (27.3)	20.5–35
High school	104 (29.6)	25 (24)	16.6–32.9
College	24 (6.8)	8 (33.3)	17.2–53.2
Occupation, *n* (%)			
Student	107 (30.5)	26 (24.3)	16.9–33
Household	97 (27.6)	17 (17.5)	11–26
Farmer	58 (16.5)	20 (34.5)	23.2–47.2
Public employee	38 (10.8)	11 (28.9)	16.5–44.5
Unemployed	16 (4.6)	2 (12.5)	2.7–34.4
Educator	12 (3.4)	5 (41.7)	18–68.8
Merchant	7 (2)	2 (28.6)	6.5–64.8
Other	16 (4.6)	4 (25)	9.1–49.1
Pregnancy, yes (%)	4 (2.1)	1 (25)	2.8–71.6
Domicile: municipality, *n* (%)			
Cajigal	236 (67.2)	63 (26.7)	21.4–32.6
Valdez	79 (22.5)	13 (16.5)	9.6–25.8
Cruz Salmerón Acosta	36 (10.3)	11 (30.6)	17.4–46.7
Domicile: parish, *n* (%)			
El Paujil	154 (43.9)	44 (28.6)	21.9–36.1
Yaguaraparo	82 (23.4)	19 (23.2)	15.1–33.1
Cristóbal Colón	79 (22.5)	13 (16.5)	9.6–25.8
Chacopata	36 (10.3)	11 (30.6)	17.4–46.7
Years of residence in domicile, median (IQR), years	12 (5–22)		
Years of residence in domicile, *n* (%)			
< 5 years	78 (22.2)	16 (20.5)	12.7–30.4
5–10 years	82 (23.4)	25 (30.5)	21.3–41
> 10 years	191 (54.4)	46 (24.1)	18.4–30.5
Previous malaria, *n* (%)			
No	117 (33.3)	30 (25.6)	18.4–34.1
Yes	234 (66.7)	57 (24.4)	19.2–30.2
No. of total episodes, median (IQR)	3 (2–7)		
No. of episodes in total, *n* (%)			
1–5	159 (67.9)	36 (22.6)	16.7–29.6
6–10	49 (20.9)	13 (26.5)	15.8–40
≥ 11	26 (11.1)	8 (30.8)	15.8–49.8
Parasite of last episode, *n* (%)			
*P. vivax*	165 (70.5)	41 (24.8)	18.7–31.8
*P. falciparum*	9 (3.8)	3 (33.3)	10.4–65.2
* P. vivax*/*P. falciparum*	16 (6.8)	3 (18.8)	5.6–42.1
Unknown	44 (18.8)	10 (22.7)	12.3–36.6
Adherence to treatment of last episode, *n* (%)			
Yes	151 (64.5)	33 (21.9)	15.8–28.9
No	83 (35.5)	24 (28.9)	20–39.3
Positive RDT, yes (%)	1 (0.3)		
Thick and thin blood smear, yes (%)	1 (0.3)		
Positive PCR, yes (%)	87 (24.8)		20.5–29.5
Parasite, *n* (%)			
* P. vivax*	64 (73.6)		
* P. falciparum*	8 (9.2)		
* P. vivax*/*P. falciparum*	13 (14.9)		
* P. malaria*	2 (2.3)		

*IQR* interquartile range, *PCR* polymerase chain reaction, *CI* confidence interval

### Prevalence of asymptomatic malaria

**Fig. 3:**
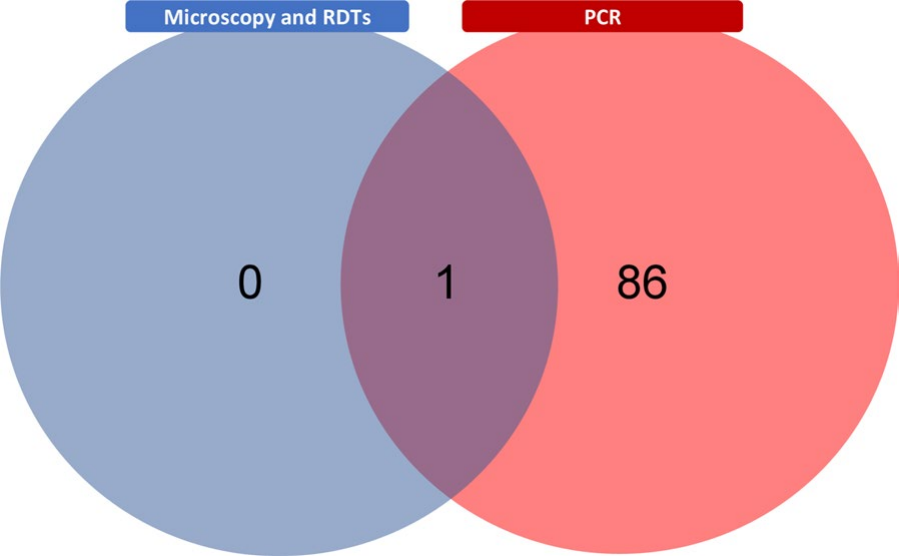
Prevalence of asymptomatic malaria. Venn diagram illustrating the overlap of asymptomatic individuals who tested positive using RDTs, microscopy, and molecular techniques (PCR). *RDTs* rapid diagnostic tests, *PCR* polymerase chain reaction

The prevalence of asymptomatic malaria was 0.3% (*n* = 1/351) as determined by both microscopy and RDT, while by PCR was 24.8% (*n* = 87/351, 95.0% CI = 20.5–29.5), including *P. vivax* (73.6%, *n* = 64), *P. falciparum* (9.2%, *n* = 8), mixed *P. vivax*/*P. falciparum* infection (14.9%, *n* = 13), and *Plasmodium malariae* (2.3%, *n* = 2) asymptomatic cases (Fig. 3).

Among these individuals with asymptomatic malaria (PCR+), the highest prevalences were observed in individuals aged over 15 years (27.1%, 95.0% CI = 21.6–33.1), males (28.3%, 95.0% CI = 21.7–35.6), individuals with a college (14 years) education (33.3%, 95.0% CI = 17.2–53.2), and educators (41.7%, 95.0% CI = 18–68.8). The rural community with the highest prevalence was Chacopata (30.6%, 95.0% CI = 17.4–46.7), followed by El Paujil (28.6%, 95.0% CI = 21.9–36.1), Yaguaraparo (23.2%, 95.0% CI = 15.1–33.1), and Cristobal Colon (16.5%, 95.0% CI = 9.6–25.8). Other prevalences in individuals with asymptomatic malaria as detected by PCR are presented in Table 1.

No statistically significant differences were observed between the sociodemographic characteristics and malaria history of individuals with asymptomatic malaria (PCR+) and controls (Table 2).

**Table 2:** Sociodemographic characteristics and malaria history of individuals with asymptomatic malaria by PCR and controls

	PCR+ (*n* = 87, 24.8%)	PCR– (*n* = 264, 75.2%)	*P* value
Age, median (IQR), years	27 (12–44)	23 (12–41)	0.216^*^
Age group, *n* (%)			0.339^†^
< 5 years	5 (5.7)	25 (9.5)	
5–15 years	20 (23)	72 (27.3)	
> 15 years	62 (71.3)	167 (63.3)	
Sex, *n* (%)			0.165^†^
Female	42 (48.3)	150 (56.8)	
Male	45 (51.7)	114 (43.2)	
Race, *n* (%)			0.687^†^
Mestizo	52 (59.8)	163 (61.7)	
Black	29 (33.3)	89 (33.7)	
White	6 (6.9)	12 (4.5)	
Level of education, *n* (%)			0.39^†^
None	15 (17.2)	65 (24.6)	
Primary school	39 (44.8)	104 (39.4)	
High school	25 (28.7)	79 (29.9)	
College	8 (9.2)	16 (6.1)	
Occupation, *n* (%)			0.237^†^
Student	26 (29.9)	81 (30.7)	
Household	17 (19.5)	80 (30.3)	
Farmer	20 (23)	38 (14.4)	
Public employee	11 (12.6)	27 (10.2)	
Unemployed	2 (2.3)	14 (5.3)	
Educator	5 (5.7)	7 (2.7)	
Merchant	2 (2.3)	5 (1.9)	
Other	4 (4.6)	12 (4.5)	
Pregnancy, yes (%)	1 (2.4)	3 (2)	1^‡^
Domicile: municipality, *n* (%)			0.132^†^
Cajigal	63 (72.4)	173 (65.5)	
Valdez	13 (14.9)	66 (25)	
Cruz Salmerón Acosta	11 (12.6)	25 (9.5)	
Domicile: parish, *n* (%)			0.181^†^
El Paujil	44 (50.6)	110 (41.7)	
Yaguaraparo	19 (21.8)	63 (23.9)	
Cristóbal Colón	13 (14.9)	66 (25)	
Chacopata	11 (12.6)	25 (9.5)	
Years of residence in domicile, median (IQR), years	12 (6–25)	12 (5–21)	0.229^*^
Years of residence in domicile, *n* (%)			0.326^†^
< 5 years	16 (18.4)	62 (23.5)	
5–10 years	25 (28.7)	57 (21.6)	
> 10 years	46 (52.9)	145 (54.9)	
Previous malaria, *n* (%)			0.793^†^
No	30 (34.5)	87 (33)	
Yes	57 (65.5)	177 (67)	
No. of total episodes, median (IQR)	3 (2–7)	3 (2–7)	0.817^*^
No. of episodes in total, *n* (%)			0.619^†^
1–5	36 (63.2)	123 (69.5)	
6–10	13 (22.8)	36 (20.3)	
≥ 11	8 (14)	18 (10.2)	
Parasite of last episode, *n* (%)			0.861^†^
* P. vivax*	41 (71.9)	124 (70.1)	
* P. falciparum*	3 (5.3)	6 (3.4)	
* P. vivax*/*P. falciparum*	3 (5.3)	13 (7.3)	
Unknown	10 (17.5)	34 (19.2)	
Adherence to treatment of last episode, *n* (%)			0.229^†^
Yes	33 (57.9)	118 (66.7)	
No	24 (42.1)	59 (33.3)	

*IQR* interquartile range, *PCR* polymerase chain reaction, *CI* confidence interval

^*^Mann–Whitney U test

^†^Pearson’s Chi-square test

^‡^Fisher’s exact test

### *Plasmodium* species according to the selected rural community

*P. vivax* was the most prevalent species in all four selected rural communities. Yaguaraparo community presented the highest prevalence of mixed *P. vivax*/*P. falciparum* infections (31.6%). Additionally, *P. malariae* was detected in Cristóbal Colón (7.7%) and Yaguaraparo (5.3%) communities (Table 3).

**Table 3:** *Plasmodium* species by selected rural community

	El Paujil (*n* = 44, 50.6%)	Cristóbal Colón (*n* = 13, 14.9%)	Yaguaraparo (*n* = 19, 21.9%)	Chacopata (*n* = 11, 12.6%)
Parasite, *n* (%)				
* P. vivax*	35 (79.5)	10 (76.9)	12 (63.2)	7 (63.6)
* P. falciparum*	5 (11.4)	2 (15.4)	0 (0)	1 (9.1)
* P. vivax*/*P. falciparum*	4 (9.1)	0 (0)	6 (31.6)	3 (27.3)
* P. malariae*	0 (0)	1 (7.7)	1 (5.3)	0 (0)

## Discussion

This study found that the prevalence of asymptomatic malaria in Sucre was 24.8% (95% CI = 20.5–29.5), significantly higher than the 8.0% reported in Cajigal municipality, Sucre state, in 2003 [2]. This difference could be attributed to the unprecedented increase in symptomatic malaria cases in Venezuela, including Sucre state [32]. Compared to the prevalence of asymptomatic malaria in countries with moderate to low transmission rates, such as Brazil (2.4–3.4%), Colombia (4.0%), and Ecuador (10.0%), the prevalence observed in this study is markedly higher [33–36]. However, it is lower than in malaria hyper-endemic areas such as Ghana, Senegal, and Tanzania (70.3–73.0%) [16, 18, 20, 37]. This study also noted a variation in parasite species prevalence, highlighting the influence of geographic location and endemicity levels [38]. Notably, the Venezuelan Ministry of Health does not currently provide estimates on the prevalence of asymptomatic malaria cases within the country.

Interestingly, this study identified a higher prevalence of asymptomatic malaria among individuals aged over 15 years, similar to findings in studies conducted in Bangladesh [39], Tanzania [20], and Ghana [40]. This suggests the development of acquired immunity that correlates with age and parasite exposure. In regions of moderate endemicity, parasite density decreases with age, with the paediatric population being the primary reservoir for sexual forms of the parasite. In low-endemicity settings like this study site, immunity appears to be age-dependent, resulting in a higher prevalence among older age groups [38–41]. This research demonstrated a higher proportion of asymptomatic malaria cases among individuals older than 15 years, emphasizing the need for control strategies that consider endemicity levels and age groups. The prevalence of asymptomatic individuals was higher among those who had resided in the region for longer than five years, which correlates with exposure-related immunity, where individuals frequently exposed to parasites for prolonged periods develop an immunological memory that suppresses infection, even in areas of low transmission [42–44]. Unlike other studies [45], this research did not find a correlation between asymptomatic malaria and a history of the disease, indicating the potential influence of other factors such as genetic susceptibilities or local variations in vector ecology. Due to the lack of official reports with detailed data, it is not possible to compare the profile of asymptomatic and symptomatic malaria cases. However, the sociodemographic profile of symptomatic cases varies from state to state throughout the country. For example, in Bolívar state, young miners are more frequently found in mining regions among malaria cases [46]. In contrast, in Sucre state, mining regions are scarce, and malaria is associated with agricultural activities, unplanned urban growth, and unfavourable socioeconomic conditions [5].

Molecular techniques such as PCR are necessary because of the low sensitivity of RDTs and microscopy when parasite load is low [47]. Other studies report that the sensitivity of RDTs to detect asymptomatic malaria cases was 59.4% in Senegal, and 43.8% in Ghana [15], with microscopy sensitivity of 56.4% (whereas PCR has specificity and sensitivity of 100.0%) [48]. In this study, out of 351 asymptomatic malaria cases, RDTs and microscopy detected only one positive case. At the same time, PCR identified 81 individuals with submicroscopic infections, denoting a sensitivity of less than 1.0%, comparable with a study in Ecuador [36]. This highlights the importance of using sensitive diagnostic methods, especially in low-endemicity areas, to accurately identify asymptomatic cases, particularly *P. vivax* infections that significantly contribute to the gametocyte reservoir [49], posing a significant challenge for disease eradication.

Although historically the prevalence of *P. malariae* in Venezuela has been < 1.0% [50], there are few studies in Venezuela on this species, and none report its identification in asymptomatic infection. Here, it was detected two asymptomatic individuals infected with *P. malariae*, highlighting the need for surveillance and molecular characterization of this species, given the limitation of specific rapid tests. Maximum parasite counts are often low compared to those of individuals infected with *P. falciparum* or *P. vivax*. This is due to several factors, including the lower number of merozoites produced per erythrocyte cycle and the parasite’s preference to develop in older erythrocytes [51]. Consequently, low parasitaemia limits its detection by microscopy. In Cameroon, a significant contribution of *P. malariae* to the high malaria transmission rate has recently been documented, with 2.5% identified as mono-infected [52]. Another study in the same country reported a 12.0% prevalence of *P. malariae* in asymptomatic individual samples [53]. Therefore, initiatives are needed to target this parasite species in both symptomatic and asymptomatic individuals to understand its role in transmission [54].

The impact of climate change on malaria transmission remains a topic of debate. While some studies have reported a correlation between rising temperatures and increased malaria incidence in selected sub-Saharan African countries [55], others have projected a reduced malaria burden in the western sub-region of West Africa and negligible effects in the eastern sub-region [56].

Additionally, research has shown that even small temperature changes may reduce or block the transmission potential of certain vectors, such as *Anopheles stephensi* and *Anopheles gambiae*. Thus, rather than increasing malaria risk, current and future warming may reduce transmission potential in regions with existing high transmission [57].

In Venezuela, the inter-annual variability in malaria cases has shown a correlation with sea surface temperatures, particularly on timescales of 3–6 years. Malaria cases have also increased approximately one year after an El Niño event, emphasizing the role of interannual climate variability in malaria epidemics. Local rainfall, particularly late-season precipitation, mediated the impact of the El Niño Southern Oscillation on malaria. However, the relationship between climate and malaria is complex, transient, and region-specific, with variations in intensity based on geographic area and parasite species [58]. Climate change could potentially amplify the effects of El Niño Southern Oscillation, leading to alterations in rainfall patterns that affect mosquito breeding and malaria transmission, thus influencing the prevalence of asymptomatic malaria. Nevertheless, the precise impact of climate change on asymptomatic malaria remains unknown.

This study has several limitations. First, because individuals were not followed up, it is not known whether they were in a pre-symptomatic period of the disease. Second, it included only four communities in Sucre. However, this study’s random sampling provides valuable information on the prevalence and characteristics of asymptomatic malaria in Sucre state. Finally, it was unable to calculate the parasitaemia that would have allowed us to establish a clinical correlation. Future research is needed not only in Sucre state but also in other regions of the country, given the limited availability of information on asymptomatic malaria.

## Conclusions

This study documents a high prevalence of asymptomatic malaria in four communities of Sucre state, a state that contributes significantly to the malaria burden in Venezuela, compared to previous regional reports. Less than 1.0% of asymptomatic malaria cases were diagnosed using RDTs and microscopy, highlighting the necessity of molecular techniques for accurate diagnosis and effective treatment of these cases.

## Supplementary Information

Supplementary Material 1

Supplementary Material 2

Supplementary Material 3

## Data Availability

All data and materials in this article are included in the manuscript.

## Abbreviations

API: Annual parasite index
MSAT: Mass screening and treatment
RDTs: Rapid diagnostic tests
PCR: Polymerase chain reaction
SD: Standard deviation
IQR: Interquartile range
