# Supplementary material for: Prevalence and epidemiological characteristics of asymptomatic malaria in Sucre, Venezuela: a 2022 cross-sectional study
Source: Malar J. 2025 Apr 13;24:120. doi: 10.1186/s12936-025-05356-z (PMC11993942; doi:10.1186/s12936-025-05356-z)
Supplement: Supplementary file 1 — Supplementary Material 1 [file 12936_2025_5356_MOESM1_ESM.docx]

**Supplementary Data 1.** Questionnaire
