# Supplementary material for: Prevalence and epidemiological characteristics of asymptomatic malaria in Sucre, Venezuela: a 2022 cross-sectional study
Source: Malar J. 2025 Apr 13;24:120. doi: 10.1186/s12936-025-05356-z (PMC11993942; doi:10.1186/s12936-025-05356-z)
Supplement: Supplementary file 2 — Supplementary Material 2 [file 12936_2025_5356_MOESM2_ESM.docx]

**Supplementary Data 2.** Names and sequences of primers

| **Names of primers** | **Sequences of primers** |
| --- | --- |
| rPLU5 | 5’-CCTGTTGTTGCCTTAAACTTC-3’ |
| rPLU6 | 5’-TTAAAATTGTTGCAGTTAAAACG-3’ |
| rFAL-F | 5’-CTTTTGAGAGGTTTTGTTACTTTGAGTAA-3’ |
| rFAL-R | 5’-TATTCCATGCTGTAGTATTCAAACAAAA-3’ |
| rVIV-F | 5’-ACGCTTCTAGCTTAATCCACATAACT-3’ |
| rVIV-R | 5’-ATTTACTCAAAGTAACAAGGACTTCCAAGC-3’ |
| rMAL-F | 5’-ATAACATAGTTGTACGTTAAGAATAACCGC-3’ |
| rMAL-R | 5’-AAAATTCCCATGCATAAAAAATTATACAAA-3’ |
