# Supplementary material for: Prevalence and epidemiological characteristics of asymptomatic malaria in Sucre, Venezuela: a 2022 cross-sectional study
Source: Malar J. 2025 Apr 13;24:120. doi: 10.1186/s12936-025-05356-z (PMC11993942; doi:10.1186/s12936-025-05356-z)
Supplement: Supplementary file 3 — Supplementary Material 3 [file 12936_2025_5356_MOESM3_ESM.docx]

**Supplementary Data 3.** Showing the expected band size for *Plasmodium* genus and species

| **Genus and species** | **Name of primers** | **Expected band size (bp)** |
| --- | --- | --- |
| *Plasmodium* genus | rPLU5 and rPLU6 | 1200 |
| *Plasmodium falciparum* | rFAL-F and rFAL-R | 205 |
| *Plasmodium vivax* | rVIV-F and rVIV-R | 120 |
| *Plasmodium malariae* | rMAL-F and rMAL-R | 140 |
